# Supplementary material for: COVID-19 vaccine uptake and hesitancy survey in Northern Ireland and Republic of Ireland: Applying the theory of planned behaviour
Source: PLoS One. 2021 Nov 17;16(11):e0259381. doi: 10.1371/journal.pone.0259381 (PMC8598022; doi:10.1371/journal.pone.0259381)
Supplement: Attachment — Submitted filename: Letter to the editor.docx [file pone.0259381.s001.docx]

Dr. Gavin Breslin
School of Psychology
Ulster University
Cromore Road
Coleraine

Northern Ireland

BT521SA

Email: g.breslin1@ulster.ac.uk

28/07/21

Dear Professor Anat Gesser-Edelsburg,

Thank you for considering our article entitled: ‘*COVID-19 Vaccine Uptake and Hesitancy Survey in Northern Ireland and Republic of Ireland: Applying the Theory of Planned Behaviour’* (PONE-D-21-16573) for publication in PLOS ONE

We write to confirm that the authors have read the reviewers’ feedback, and the manuscript has been revised in light of these comments. We would like to take this opportunity to thank the reviewers for their comments, and believe the manuscript is now much improved.

As requested, we have included responses to the reviewers’ comments in this rebuttal letter, and detailed our changes in the manuscript using the tracked changes format in Microsoft Word. We have also submitted a version of the article were track changes are removed.

We hope you find our changes adequate, and our revised article suitable for publication*.*

We look forward to hearing back from you.

Yours sincerely,

Dr Gavin Breslin (corresponding author)

**Journal Requirements:**

**When submitting your revision, we need you to address these additional requirements.**

**1. Please ensure that your manuscript meets PLOS ONE's style requirements, including those for file naming. The PLOS ONE style templates can be found at**

**https://journals.plos.org/plosone/s/file?id=wjVg/PLOSOne_formatting_sample_main_body.pdf and**

[**https://journals.plos.org/plosone/s/file?id=ba62/PLOSOne_formatting_sample_title_authors_affiliations.pdf**](https://journals.plos.org/plosone/s/file?id=ba62/PLOSOne_formatting_sample_title_authors_affiliations.pdf)

Thank you for providing this information, we have updated the article to reflect the style of formatting required.

**2. Thank you for including your ethics statement: 'Ethical approval was granted by Ulster University. All participants provided informed consent and were free to withdraw at any time. No personal identifying data was collected to ensure confidentiality.'**

**a. Please amend your current ethics statement to include the full name of the ethics committee/institutional review board(s) that approved your specific study.**

The full name of the committee has been added.

**b. Once you have amended this/these statement(s) in the Methods section of the manuscript, please add the same text to the “Ethics Statement” field of the submission form (via “Edit Submission”).**

Matthew I have added in the article can you add as I don’t have access

**For additional information about PLOS ONE ethical requirements for human subjects research, please refer to** [**http://journals.plos.org/plosone/s/submission-guidelines#loc-human-subjects-research**](http://journals.plos.org/plosone/s/submission-guidelines#loc-human-subjects-research)

Thank you for this additional guidance.

**3. Please provide additional details regarding participant consent. In the ethics statement in the Methods and online submission information, please ensure that you have specified what type you obtained (for instance, written or verbal, and if verbal, how it was documented and witnessed). If your study included minors, state whether you obtained consent from parents or guardians. If the need for consent was waived by the ethics committee, please include this information.**

Participants were provided with a description of the aims and rationale for the study and were asked to provide informed consent before taking part in the survey. Participants could not take the survey without consenting.

**4. PLOS requires an ORCID iD for the corresponding author in Editorial Manager on papers submitted after December 6th, 2016. Please ensure that you have an ORCID iD and that it is validated in Editorial Manager. To do this, go to ‘Update my Information’ (in the upper left-hand corner of the main menu), and click on the Fetch/Validate link next to the ORCID field. This will take you to the ORCID site and allow you to create a new iD or authenticate a pre-existing iD in Editorial Manager. Please see the following video for instructions on linking an ORCID iD to your Editorial Manager account: https://www.youtube.com/watch?v=_xcclfuvtxQ**

This has been completed on Editoroial Manager. Dr Gavin Breslin’s ORCID ID is: 0000-0003-2481-0860.

**[Note: HTML markup is below. Please do not edit.]**

**Reviewers' comments:**

**Reviewer's Responses to Questions**

**Comments to the Author**

**1. Is the manuscript technically sound, and do the data support the conclusions?**

**The manuscript must describe a technically sound piece of scientific research with data that supports the conclusions. Experiments must have been conducted rigorously, with appropriate controls, replication, and sample sizes. The conclusions must be drawn appropriately based on the data presented.**

**Reviewer #1: Partly**

**Reviewer #2: Yes**

**________________________________________**

**2. Has the statistical analysis been performed appropriately and rigorously?**

**Reviewer #1: Yes**

**Reviewer #2: Yes**

**________________________________________**

**3. Have the authors made all data underlying the findings in their manuscript fully available?**

**The PLOS Data policy requires authors to make all data underlying the findings described in their manuscript fully available without restriction, with rare exception (please refer to the Data Availability Statement in the manuscript PDF file). The data should be provided as part of the manuscript or its supporting information, or deposited to a public repository. For example, in addition to summary statistics, the data points behind means, medians and variance measures should be available. If there are restrictions on publicly sharing data—e.g. participant privacy or use of data from a third party—those must be specified.**

**Reviewer #1: Yes**

**Reviewer #2: Yes**

**________________________________________**

**4. Is the manuscript presented in an intelligible fashion and written in standard English?**

**PLOS ONE does not copyedit accepted manuscripts, so the language in submitted articles must be clear, correct, and unambiguous. Any typographical or grammatical errors should be corrected at revision, so please note any specific errors here.**

**Reviewer #1: Yes**

**Reviewer #2: Yes**

**________________________________________**

**5. Review Comments to the Author**

**Please use the space provided to explain your answers to the questions above. You may also include additional comments for the author, including concerns about dual publication, research ethics, or publication ethics. (Please upload your review as an attachment if it exceeds 20,000 characters)**

**Reviewer #1: In this manuscript by Cavanagh and colleagues, the authors used the theory of planned behavior to examine COVID-19 vaccine uptake. This is a timely article as many countries are struggling with COVID-19 vaccine uptake during this pandemic and this article has the potential to provide recommendations and suggestions on addressing vaccine hesitancy.**

Thank you for the kind comments and to read that the article is timely and of international interest in addressing vaccine hesitancy.

**1. The "Aims of the Study" section is a bit confusing because it is a list of aims and isn't clearly connected to the introduction section. It would be helpful if the authors, clearly stated gaps in existing literature and how study aims will fill existing knowledge gaps.**

The aims of the study section has been adjusted to reflect that there are gaps in the literature. Page 8 line 187-198

**2. The last aim mentions "to consider confidence in giving the COVID-19 vaccine to children..." however, none of the measures focus on vaccine acceptance for children, and there is no mention of this aim in the results or discussion. If this aim is not included in the current paper, then it should be removed from that section.**

The aim has been deleted.

**3. Participant demographics should be in the results section.**

Participant demographics have been moved to the results section, Page 9 281-284.

**4. "Cross-sectional online survey" is not enough information on the research design. Overall, the methods section is missing a lot of information. For example, if social media platforms were used, additional information on the specific platforms used is needed, along with how the study was advertised on these platforms, such as were flyers posted in specific groups? Did the study staff pay social media platforms to advertise the study to specific populations? This section also needs information on incentives provided to participants.**

The Research Design section has been updated with the above recommendations. Page 9 205-208.

**5. Overall, the discussion section seems more like an extensions of the introduction and provides little information about how the results from this study relate to previous studies. Additionally, the authors stated how the aims of this study was to provide suggestions, but suggestions seemed based off of previous literature instead of study results.**

Sentences have been added to the discussion to show how findings informs recommendations. The recommendations using factors from the Theory of Planned behaviour are now articulated in the dicussion section. Page 19 line 356-358, 363-364, 390-391.

**Reviewer #2: Preface**

**The manuscript is well written, and the study well-conducted I very much appreciated the not easy field of why there is reluctance or hesitation to vaccination programs and especially in some age groups also relates to changes in the patient-doctor relationship but also in the communication of vaccination policy by the ministries or political representatives of the different countries in the world.**

**The Scottish school has previously addressed this issue as well for other vaccinations.**

**Kennedy C, Gray Brunton C, Hogg R. 'Just that little bit of doubt': Scottish parents', teenage girls’, and health professionals' views of the MMR, H1N1 and HPV vaccines. Int J Behav Med. 2014 Feb;21(1):3-10. DOI: 10.1007/s12529-013-9356-4. PMID: 24198038.**

Thank you for bringing this article by kennedy et al (2014) to our attention. We have included within the introduction on page 6 and 7 line 140-145.

**Introduction**

**It is too rhetorical to put the history of SAR Covid 2 infections and makes the manuscript banal I would immediately introduce the concept of vaccination also citing other examples in which in the last decade in the world and some countries, it is especially in Europe there is a certain reluctance even towards vaccinations already well known as measles, this reluctance is also encountered in booster.**

**Please see the following manuscripts**

**Hilton S, Patterson C, Smith E, Bedford H, Hunt K. Teenagers' understandings of and attitudes towards vaccines and vaccine-preventable diseases: a qualitative study. Vaccine. 2013;31(22):2543-2550. doi:10.1016/j.vaccine.2013.04.023**

The study by Hilton has been incorporated into the introduction of the article on page 6.

**The data collection methodology is corrected, and the results are well depicted.**

Thank you for your positive comment.

**The statistical analysis was well conducted. Authors should also consider whether to report a multivariate analysis.**

A multiple linear regression analysis was calculated to incorporate the predictor of vaccine intention Page 17 line 336.

**In the debate, I would gently stress the fact that we do not yet know the events that the mutation of the virus will determine on its effectiveness and that it is better to explain the truth to those who must undergo vaccination than to be sure of its effectiveness. Too many times we have hesitated in the methods of communication that are not entrusted to the clinicians but politicians and that the patient finds as a reference preferably the family doctor or pediatrician rather than the minister of health.**

We have incorporated these points in the discussion, page 21 line 403-405, also see page 21 407-411

**The authors could add that in the future they will evaluate adherence to vaccination with multiple doses and compliance with other vaccinations that are added to those recommended in the different age groups (guys and elderly and high-risk group)**

This has been included page 23 line 456-458

**________________________________________**

**6. PLOS authors have the option to publish the peer review history of their article (what does this mean?). If published, this will include your full peer review and any attached files.**

**If you choose “no”, your identity will remain anonymous but your review may still be made public.**

**Do you want your identity to be public for this peer review? For information about this choice, including consent withdrawal, please see our Privacy Policy.**

**Reviewer #1: No**

**Reviewer #2: Yes: Paola Di Carlo**
